# The ResQu Index: A new instrument to appraise the quality of research on birth place

**DOI:** 10.1371/journal.pone.0182991

**Published:** 2017-08-10

**Authors:** Saraswathi Vedam, Chris Rossiter, Caroline S. E. Homer, Kathrin Stoll, Vanessa L. Scarf

**Affiliations:** 1 Centre for Midwifery, Child and Family Health, Faculty of Health, University of Technology Sydney, Broadway, NSW, Australia; 2 UBC Midwifery Faculty of Medicine, University of British Columbia, University Boulevard, Vancouver, BC, Canada; Univesity of Iowa, UNITED STATES

## Abstract

**Objective:**

Place of birth is a known determinant of health care outcomes, interventions and costs. Many studies have examined the maternal and perinatal outcomes when women plan to give birth in hospitals compared with births in birth centres or at home. However, these studies vary substantially in rigour; assessing their quality is challenging. Existing research appraisal tools do not always capture important elements of study design that are critical when comparing outcomes by planned place of birth. To address this deficiency, we aimed to develop a reliable instrument to rate the quality of primary research on maternal and newborn outcomes by place of birth.

**Study design:**

The instrument development process involved five phases: 1) generation of items and a weighted scoring system; 2) content validation via a quantitative survey and a modified Delphi process with an international, multi-disciplinary panel of experts; 3) inter-rater consistency; 4) alignment with established research appraisal tools; and 5) pilot-testing of instrument usability.

**Results:**

A Birth Place Research Quality Index (ResQu Index) was developed comprising 27 scored items that are summed to generate a weighted composite score out of 100 for studies comparing planned place of birth. Scale content validation indices were .89 for clarity, .94 for relevance and .90 for importance. The Index demonstrated substantial inter-rater consistency; pilot-testing confirmed feasibility and user-friendliness.

**Conclusion:**

The ResQu Index is a reliable instrument to evaluate the quality of design, methods and interpretation of reported outcomes from research about place of birth. Higher-scoring studies have greater potential to inform evidence-based selection of birth place by clinicians, policy makers, and women and their families. The Index can also guide the design of future research on place of birth.

## Introduction

The selection of birth place has important implications for the health and wellbeing of women and newborns, as well as for health services planning and healthcare costs [1–3]. Decisions about place of birth are largely based on the preferences of women and their families, but are also influenced by the availability of services, equipment and providers in their communities, and their understanding of the available scientific evidence [4, 5]. Women’s preferences about place of birth are most often based on provider or peer recommendations, or information gained via the Internet [6–11]. However, these sources do not always provide unbiased information about benefits and risks associated with different birth settings.

Evidence on the relative safety of different places of birth is complex and frequently controversial [12]. Several studies [13–19] have found reduced obstetric interventions and optimal outcomes among healthy women who planned to give birth at home or a birth centre under the care of midwives. In some high-income countries, where maternity care is integrated across birth settings, researchers have concluded that there are no significant differences between birth places in morbidity or mortality for newborns [15, 16, 19] and/or that the absolute risks of mortality are extremely low [13, 14]. Other investigators have reported a significant increase in adverse perinatal outcomes related to planned home births, especially where skilled birth attendants are not universally integrated into regional health systems, or in population-based studies that include at-risk pregnancies [20–22].

Policy makers and clinician leaders have responded to the research with similar discordance. Some clinical guidelines and policy statements in high-income countries support access to midwife-led care in birth centres and home births as cost-effective options for women with uncomplicated pregnancies [23–26]. Other professional organisations have issued statements questioning the evidence basis for support of women’s choice of birth place, and stating that hospital birth is the only setting that assures safe outcomes [27, 28]. In low- and middle-income countries, these latter views have led to policy initiatives and incentives towards universal institutional birth [29–31].

Inconsistency in the design and quality of research on place of birth underpins the difficulty of crafting universally acceptable recommendations for service provision across birth settings. Further, the interpretation and dissemination of research findings can be subject to publication and critical bias, and the influence of professional viewpoints and culture [12, 32].

Accurate information about relative and absolute risk is vital to inform women and their families, clinicians and health administrators as they work together to select optimal birth places within the context of a person-centred care plan and regional resources. However, without a reliable means to assess the quality of the evidence, it is not possible to interpret the best available data and its implications for the safety of women and infants. This study was designed to address the need for a standardised system for evaluating research on the safety of birth place. It aimed to develop and pilot test a novel instrument for appraising the quality and rigour of research that examines the impact of place of birth on maternal and perinatal outcomes.

### Why develop an instrument specific to birth place?

While there are several valuable instruments for assessing the quality of randomised and non-randomised clinical studies [33–38], these are not always appropriate to studies of place of birth. Some rating tools are only applicable to randomised studies; others focus on the extent to which non-randomised studies emulate randomised trials (e.g. by reducing selection bias) [34, 38–41]. However, birth place represents an intervention that is difficult to evaluate through randomised trials. Women are not inclined to relinquish their choice of birth place and participate in trials that present major practical and ethical limitations [42]. Another limitation of conventional rating instruments in this context is that, unlike many medical conditions where the impact of interventions on recovery or amelioration can be studied, pregnancy, labour and birth are not diseases or injuries (although they may be accompanied by morbidity). A woman may have multiple pregnancies, but each birth is a unique and finite event. Therefore instruments focused on longer-term recovery may not be relevant to studies on labour and birth, e.g. items on proportion of participants followed-up over time to ascertain whether symptoms have recurred.

When comparing outcomes across birth settings, the use of consistent definitions and inclusion criteria across cohorts, and reliable outcome measures, is imperative. A significant error in some published research on birth place is amalgamating data from unplanned home births (without skilled birth attendants) with data from planned births at home or in birth centres within integrated systems.

### Aims

Following revelations of critical flaws in some published studies [43–46], researchers developed key principles for appraising the quality of research on place of birth [45, 47–49]. Our team designed the Birth Place Research Quality Index (ResQu Index) as a quantitative scoring system based on these key principles, as well as best practices for critical appraisal of research [39, 50]. The objective of this paper is to describe the development and testing of the ResQu Index which aims to facilitate consistent assessment of the quality of research on place of birth.

## Methods

Development of the ResQu Index involved five distinct phases: 1) generating items and a weighted scoring system; 2) conducting expert content validation via a quantitative survey and a modified Delphi process; 3) testing inter-rater consistency; 4) assuring compatibility with established research quality checklists and 5) piloting the ResQu Index in a large systematic review to assess instrument usability and feasibility.

We developed this instrument to conduct an extensive systematic review of the literature on outcomes related to place of birth, within a larger research project [blinded]. A protocol was lodged with PROSPERO (CRD42016042291). Ethics clearance was not required for the instrument development.

### Phase 1: Item generation and scoring

Item generation was informed by a literature review and the principles proposed by Vedam [45], Hutton [51] and Nove [47]. Each item was selected to be consistent with other systems for appraisal of research quality [34, 35, 37, 38, 52] but adapted, when necessary, to focus on the unique aspects of comparing outcomes across birth settings.

Some items in the Index relate to rigour common to all research studies, addressing issues such as clarity of key terms, definitions of the ‘intervention’ (i.e. place of birth), integrity of data, appropriateness of sample size and selection, transparency of methods and comparability of cohorts. Other items relate specifically to studies of birth place as outlined by Vedam [49] and Nove [47], and to address issues identified as problematic in critiques of previous research in this field [43, 44, 46]. These items include identifying the timing of birth place decisions in relation to an intention-to-treat model and ensuring that studies of home birth clearly distinguish data from planned home births with skilled attendants, from data generated by unplanned home births or “free births” without professional support. The individual items need to be relatively broad to be applicable to studies of different birth settings available across various regions. However, they also need to be sufficiently focused to identify meaningful differences in the rigour of research methodology.

Each item incorporates a rubric that scores the level of quality of the study design and interpretation of findings. Item scores are summed to generate a total composite Index score for a study. Scoring options vary from a simple yes/no response to a range of potential criteria. The expert content validation process (Phase 2) evaluated both the wording of the scoring rubrics and their relative numerical value (weighting).

**Fig 1:**
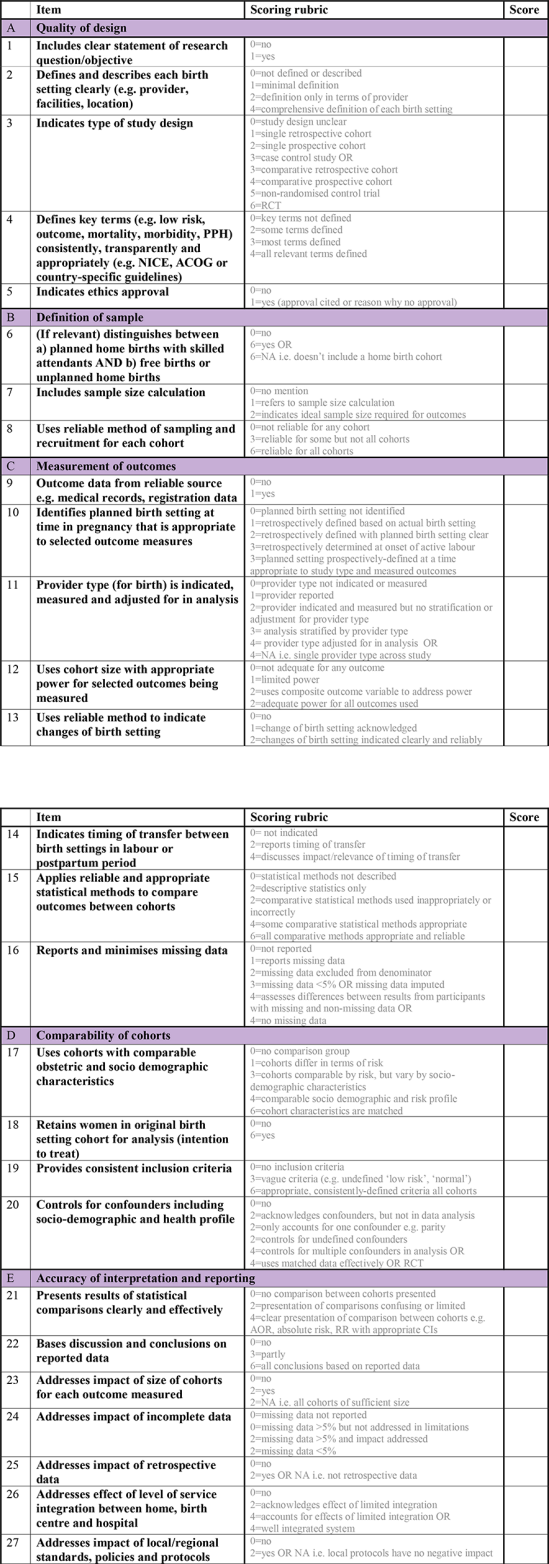
Birth Place Research Quality (ResQu) Index—Final version.

The first draft version of the ResQu Index contained 25 items, relating to five domains addressing: quality of design, sample definition, measurement of outcomes, comparability of cohorts, and accuracy of interpretation and reporting (domains indicated in Fig 1). The Index scores total to 100, with higher scores indicating higher quality. The wording and scoring scale of items in the first draft version are included under Results.

### Phase 2: Expert content validation

#### Participants

An international multidisciplinary expert panel was invited to examine the draft ResQu Index for face and content validity via a quantitative survey and a modified Delphi process involving multiple rounds of consultation. The panel constituted both content experts and potential users of the Index, both of whom should be included in a content validation [53, 54]. In line with best practice for expert panel review, the invited panel of 42 experts spanned a range of perspectives in terms of profession, expertise, country of residence/practice, and acknowledged attitudes about place of birth [53, 54]. In total, 21 experts completed the validation process.

The panel included 15 academics, 12 researchers, 8 midwives, 6 perinatal epidemiologists, 4 statisticians, 2 policy makers and one each of consumer, nurse and obstetrician (more than one response was possible). Their areas of expertise included: research on birth place (16 experts), planned home birth (13), research methodology/study design (11), midwifery practice (11), evidence-based practice (10), health services (10), appraisal of evidence (10), public health (9), hospital maternity care (7), birth centres (7), health systems (5), statistics (5), population-based services (4), patient-oriented outcomes (4), global health (3). Two experts indicated professional interest in ‘transfer’ and one each ‘law or policy’, ‘nursing practice’ and ‘medical practice’. Anonymous response was possible, but twelve of the 21 experts supplied their names. All supplied the country/region in which they were based, including North America (11), Australia (7), United Kingdom (2), and the Netherlands (1). Several indicated that they also worked in a range of other countries or regions.

#### Procedure

Panel members were contacted using publicly-available contact details, and participation was voluntary. We emailed the panel of 42 experts explaining the background and purpose of the Index, and inviting them to participate in the expert review process. We outlined the conceptual basis and scope of the Index [53] and explained how the process differed from content validation for instruments assessing skills or attitudes.

The content validation took place during June 2016, online via Survey Gizmo. Experts considered each item on the first draft version for clarity, importance and relevance, rating each aspect on a four-point ordinal scale from ‘very clear/important/relevant’ (1) to ‘not clear/important/relevant’ (4). In addition to the quantitative rating process, they were invited to provide written feedback on the wording and scoring rubric for each item, to suggest non-essential items that might be omitted, and to comment on individual items and/or the overall purpose, utility and scope of the draft Index [55].

Following expert review, several items were amended in line with the experts’ quantitative responses and qualitative feedback (S1 and S2 Tables give examples of this process). Two team members used the updated version to assess 41 studies, and made small adjustments in wording to arrive at a second draft version. The second draft version was re-circulated for review and approval to the expert panel.

To assess utility and user-friendliness, we invited the same experts to beta-test the second draft ResQu Index, by supplying them with two published articles on birth place to assess. The articles were selected to reflect different settings, methodologies, and eras. They were from two different countries, one a relatively recent study comparing outcomes from freestanding midwifery units with hospital obstetric units and the other a pre-2000 study comparing home births and hospital births. Four completed this beta-testing process, including two who did not participate in the first stage of content validation. Responses and recommendations were incorporated into the final draft before pilot testing.

#### Data analysis

Responses from the online survey were imported into MS Excel for simple descriptive analysis. An item content validation index (I-CVI) was calculated for the three aspects (clarity, importance and relevance) of each item, being the proportion of experts that gave it a positive response (1 or 2). Items that achieved a total score above .80 for relevance were retained (except for one which scored low in importance). Those items below .80 for clarity were rephrased or modified to reflect the expert comments (S1 and S2 Tables) or discarded. The overall scale content validity index (S-CVI) was calculated for each aspect, being the average (mean) of the I-CVIs of the retained items. Polit and Beck recommend that a level of .90 for the S-CVI be used as the standard for excellent content validity using this approach [56].

### Phase 3: Inter-rater consistency and consensus

To test inter-rater reliability, two members of the research team (VS and CR) used the second draft version of the ResQu Index (revised but still 25 items) to independently rate 20 studies. Stemler [57] describes different purposes and methods for measuring inter-rater reliability. We used Spearman’s *rho* coefficient to explore the *consistency* of our total scores for the articles because the scores were continuous but not necessarily normally distributed [57]. Secondly, we converted each study’s score to a simple scale of research evidence: strong (scores of 75% and above), moderate (65–74%) and weak (less than 65%). We then examined inter-rater *consensus* on the three-tier scale using Cohen’s *kappa* statistic [58]. We used SPSS version 23 to analyse inter-rater consistency and consensus.

In addition to investigating inter-rater consistency and consensus, this process enabled us to review each study’s total Index scores, with particular attention to where the two raters diverged. In conjunction with further comments from expert reviewers, this contributed to the final version of the Index (Fig 1).

### Phase 4: Research quality checklists

To ascertain comprehensiveness, the ResQu Index was compared to the taxonomy of domains developed by Deeks and colleagues [39] in their extensive evaluation of scales and checklists for assessing quality in non-randomised studies. We matched ResQu items to domains identified by these authors as critical to study quality, internal and external validity, and standard of reporting. We also compared the Index to the Cochrane tool for assessing Risk of Bias in Non-randomized Studies of Interventions (ROBINS-I) [38], which is widely used to assess research quality in systematic reviews [59].

### Phase 5: Pilot testing

As a final stage of development, two authors (VS and CR) assessed usability and feasibility by using the ResQu Index in a systematic review on maternal and perinatal outcomes related to place of birth for women at low risk of obstetric complications in high-income countries. We used the updated ResQu Index to rate 68 articles, comparing and discussing their ratings when total scores diverged. This process was done iteratively, in conjunction with the search and screening stages of the review. Having identified studies that met the eligibility criteria for the systematic review, one or both authors applied the current version of the Index.

## Results

### Item generation and scoring

The final ResQu Index contains 27 items across five domains, each rated on a numerical scale where higher values indicated higher quality with respect to the aspect of quality being measured. The items and the final scoring rubrics are shown in Fig 1. The total possible composite score is 100, to guide categorisation of studies into strong, moderate, and weak quality.

### Expert content validation

The experts’ quantitative responses about the clarity, importance and relevance of the items in the first draft version are summarised in Table 1. I-CVIs are the proportion of respondents who rated each item positively (1 or 2).

**Table 1:** Content validation indices–DRAFT ResQu items, proportion of expert panel giving a positive rating*.

	Item	Clarity	Relev-ance	Import-ance
1	**Defines and describes each birth settings clearly**	.90	1.00	1.00
2	**Type of study design**	.90	.90	.95
3	**Uses reliable and logical comparison group/s**	.76	.95	1.00
4	**Retains women in original birth setting cohort for data analysis (intention to treat)**	.95	1.00	1.00
5	**Distinguishes between** **a) planned home births with skilled attendants AND b) free births or unplanned home births** **(if home births included in study)**	.95	1.00	1.00
6	**Identifies planned place of birth at time in pregnancy that is appropriate to selected outcome measures**	.90	1.00	.95
7	**Accounts for effect of provider type**	.67	.90	.81
8	**Discriminates outcomes of care according to type of provider (as distinct from birth setting)**	.80	.81	.71
9	**Sample size powered appropriately for selected outcomes being measured**	.90	.90	.90
10	**Uses reliable method of initial sampling and recruitment for each cohort**	.71	.95	.90
11	**Provides consistent inclusion criteria for comparison groups**	.86	.95	.90
12	**Uses reliable method to track women when birth setting changes**	.90	1.00	1.00
13	**Addresses effect of level of service integration between home, birth centre and hospital**	.86	.86	.90
14	**Controls for confounders including socio-demographic and health profile of women in cohorts**	1.00	1.00	1.00
15	**Reports criteria for transfer (change of birth place)**	1.00	.90	.62
16	**Considers potential effects related to timing of transfer and delays to treatment**	.81	.95	.86
17	**Accounts for effect of mode of transfer (ambulance, private car, neonatal transport team etc.)**	.71	.62	.48
18	**Defines key terms (e.g. PPH, low risk, planned home birth, mortality, morbidity) consistently and transparently using recognised methods and definitions (e.g. NICE, RANZCOG or ACOG guidelines)**	.95	.95	.95
19	**Applies reliable statistical methods to compare cohorts, e.g. absolute risk, relative risk, confidence intervals**	.85	.95	.95
20	**Reports and minimises missing data**	.95	.95	.90
21	**Draws conclusions based on reported data**	1.00	.90	.90
22	**Acknowledges impact of lack of randomisation**	.95	.76	.76
23	**Acknowledges impact of size of cohorts for each outcome measured**	1.00	.90	.76
24	**Acknowledges impact of retrospective and/or incomplete data**	.90	.90	.81
25	**Acknowledges impact of local/regional standards, policies and protocols**	.95	.95	.71
	**S-CVI: Average (mean) of I-CVIs of retained items**	**.89**	**.94**	**.90**
	**S-CVI: Proportion of retained items with expert-rated I-CVIs of >80%**	**.82**	**1.00**	**.90**
**REVISIONS:**				
**Items 15, 17 and 22 (shaded) deleted.**				
**Items 7 and 8 collapsed into one item (item 10 Fig 1)**				
**Item 19 split into two (items 15 and 21 in Fig 1)**				
**Several items reworded in line with comments from experts in survey or correspondence**				

* Positive rating is either 1 “very clear/important/relevant” or 2 “clear/important/relevant but needs minor revision”

Table 2 summarises the experts’ quantitative responses about the scoring of items, showing the proportion of those who gave the proposed rubric for each item either 1 or 2.

**Table 2:** Content validation of scoring rubric for DRAFT ResQu items, proportion of experts who gave a positive rating*.

	Item	Scoring rubric	CVI
**1**	**Defines and describes each birth settings clearly**	**0 = no**	**0.9**
		**1 = partial definition**	
		**4 = each birth setting defined and described clearly**	
**2**	**Type of study design**	**0 = study design unclear**	**0.9**
		**1 = single retrospective cohort**	
		**2 = single prospective cohort**	
		**3 = case control study**	
		**4 = comparative retrospective cohort (2+ birth settings)**	
		**5 = comparative prospective cohort**	
		**6 = RCT**	
**3**	**Uses reliable and logical comparison group/s**	**0 = no comparison group**	**0.8**
		**2 = comparison group/s not appropriate**	
		**4 = comparison group/s appropriate**	
**4**	**Retains women in original birth setting cohort for data analysis (intention to treat)**	**0 = no**	**0.95**
		**6 = yes**	
**5**	**Distinguishes between**	**0 = no**	**0.9**
	**a) planned home births with skilled attendants AND b) free births or unplanned home births (if home births included in study)**	**6 = yes**	
	** **	**NA = doesn’t include a home birth cohort [deduct 6 marks from denominator in calculating % score]**	
**6**	**Identifies planned place of birth at time in pregnancy that is appropriate to selected outcome measures**	**0 = not identified**	**0.8**
		**1 = retrospectively defined based on actual birth setting**	
		**2 = at first booking**	
		**3 = at 36/40**	
		**4 = at onset of labour**	
**7**	**Accounts for effect of provider type**	**0 = no recognition of effect**	**0.6**
		**2 = acknowledged but not accounted for**	
		**4 = accounts fully for effect of provider**	
		**6 = compares same providers across settings**	
**8**	**Discriminates outcomes of care according to type of provider (as distinct from birth setting)**	**0 = no apparent distinction in outcomes**	**0.8**
		**1 = vague distinction**	
		**2 = clear distinction**	
**9**	**Sample size powered appropriately for selected outcomes being measured**	**0 = not adequate for any cohort**	**0.7**
		**1 = adequate for some but not all cohorts**	
		**2 = adequate for all cohorts**	
**10**	**Uses reliable method of initial sampling and recruitment for each cohort**	**0 = not reliable for any cohort**	**0.85**
		**3 = reliable for some but not all cohorts**	
		**6 = reliable for all cohorts**	
**11**	**Provides consistent inclusion criteria for comparison groups**	**0 = no definition of comparison groups**	**0.95**
		**3 = vague inclusion criteria (e.g. ‘birth centre eligible’)**	
		**6 = clearly, consistently defined criteria**	
**12**	**Uses reliable method to track women when birth setting changes**	**0 = no**	**0.9**
		**2 = some attempt to track women**	
		**4 = effective tracking method**	
**13**	**Addresses effect of level of service integration between home, birth centre and hospital**	**0 = no**	**0.7**
		**2 = acknowledges effect**	
		**4 = adequately accounts for effects of integration**	
		**6 = fully integrated system**	
**14**	**Controls for confounders including socio-demographic and health profile of women in cohorts**	**0 = no**	**0.9**
		**2 = acknowledges confounders, but not in data analysis**	
		**4 = controls for confounders in analysis**	
**15**	**Reports criteria for transfer (change of birth place)**	**0 = no**	**0.95**
		**1 = yes**	
**16**	**Considers potential effects related to timing of transfer and delays to treatment**	**0 = not addressed**	**0.7**
		**2 = reports timing of transfer**	
		**4 = analysis controls for timing of transfer**	
		**6 = analysis controls for delays to treatment**	
**17**	**Accounts for effect of mode of transfer (ambulance, private car, neonatal transport team etc.)**	**0 = no**	**0.65**
		**1 = yes**	
**18**	**Defines key terms (e.g. PPH, low risk, planned home birth, mortality, morbidity) consistently and transparently using recognised methods and definitions (e.g. NICE, RANZCOG or ACOG guidelines)**	**0 = no**	**0.9**
		**1 = some terms defined using non-recognised guidelines**	
		**2 = some terms defined using recognised guidelines**	
		**4 = all relevant terms defined using recognised guidelines**	
**19**	**Applies reliable statistical methods to compare cohorts, e.g. absolute risk, relative risk, confidence intervals**	**0 = no**	**0.9**
		**2 = uses limited statistical methods**	
		**4 = uses some statistical methods appropriately**	
		**6 = uses all statistical methods appropriately and effectively**	
**20**	**Reports and minimises missing data**	**0 = not reported**	**0.89**
		**1 = reports missing data**	
		**2 = missing data <5%**	
**21**	**Draws conclusions based on reported data**	**0 = no**	**0.9**
		**3 = partly**	
		**6 = all conclusions based on reported data**	
**22**	**Acknowledges impact of lack of randomisation**	**0 = no**	**0.9**
		**2 = yes**	
**23**	**Acknowledges impact of size of cohorts for each outcome measured**	**0 = no**	**0.9**
		**1 = partly**	
		**2 = yes**	
**24**	**Acknowledges impact of retrospective and/or incomplete data**	**0 = no**	**0.9**
		**2 = yes**	
**25**	**Acknowledges impact of local/regional standards, policies and protocols**	**0 = no**	**0.95**
		**2 = yes**	
** **	**S-CVI: Mean rating of retained items**	** **	**0.85**
**REVISIONS:**			
**Items 15, 17 and 22 (shaded) deleted.**			
**Items 7 and 8 collapsed into one item (item 10 Fig 1)**			
**Item 19 split into two (items 15 and 21 in Fig 1)**			
**Scoring from several items substantially reworded and/or reweighted in line with comments from experts in survey or correspondence, including items 3, 6, 9, 13, 16.**			

* Positive rating on scoring rubric is either 1 “very appropriate” or 2 “appropriate scoring scale but needs revision”

In addition to responding to the online survey, several experts corresponded directly with the project team, providing comments on the draft Index or the validation process, or seeking clarification. This correspondence, together with the quantitative and qualitative survey responses contributed to further revision of the Index. Accordingly, the wording and/or the relative weighting of scoring rubrics was refined, specifically to enhance clarity: three items were removed as feedback indicated that they were not perceived as relevant as others (items 15, 17 and 22 in Table 1); two new items were added (on sample size calculation and ethical research); one item was split into two (item 24); and two were collapsed (items 7 and 8) into one. Another (item 19) was split into two, to address separately the reliable use of statistical methods and the clarity of presentation of comparisons between birth places, and to increase the relative weighting of items on statistical rigour. We also amended the wording or scoring language for some items (S1 and S2 Tables).

The S-CVIs were calculated following the removal of the three items (items 15, 17 and 22), as described above. The average (mean) of the I-CVIs for the retained items was 0.89 for clarity, 0.94 for relevance and 0.90 for importance (Table 1). These levels are close to or above the recommended level for excellent content validity of .90 [56].

In relation to the proposed scoring rubric for the retained items, the average S-CVI was 0.85. Of the 22 retained items, seven received a positive rating of 80% or less. Of these, five were substantially reworded and or reweighted. The other two (items 7 and 8) were merged into one with a revised scoring rubric (Table 2).

The four experts who used the Index to beta-test its applicability in scoring the two sample articles reported positive experiences in terms of usability and acceptability. Following their comments, the wording and scoring in the final version were fine-tuned.

### Inter-rater reliability

Table 3 indicates results of inter-rater reliability testing for 20 studies. Four articles received identical scores. Another 11 were scored within five percentage points. There was a very strong positive correlation between the scores from the two raters (Spearman’s *rho* = .868, p<0.001), indicating a high degree of consistency in ratings. Comparing the two raters’ scores on the three-tier scale (strong, moderate, weak) also demonstrated substantial levels of consensus on the relative strength of the articles (*kappa* = .697, p<0.001) [57, 58].

**Table 3:** Comparison of ResQu Index scores on selected articles by two authors.

Article	Rater 1Score	Rater 2Score	Difference Rater 2 –Rater 1	Rater 1Strength of evidence	Rater 2Strength of evidence
A	70	76	6	Moderate	Strong
B	87	89	2	Strong	Strong
C	88	85	-3	Strong	Strong
D	96	95	-1	Strong	Strong
E	77	81	4	Strong	Strong
F	87	87	0	Strong	Strong
G	70	74	4	Moderate	Moderate
H	91	91	0	Strong	Strong
I	78	84	6	Strong	Strong
J	88	85	-3	Strong	Strong
K	89	80	-9	Strong	Strong
L	57	64	7	Weak	Weak
M	74	69	-5	Moderate	Moderate
N	73	78	5	Moderate	Strong
O	74	77	3	Moderate	Strong
P	89	89	0	Strong	Strong
Q	87	93	6	Strong	Strong
R	85	86	1	Strong	Strong
S	54	55	1	Weak	Weak
T	67	67	0	Moderate	Moderate

### Research quality checklist

The comparison of the ResQu Index with the domains identified by Deeks and colleagues [39] demonstrated considerable congruence between the Index and most of the Deeks domains (Table 4). The ResQu Index addresses some items in the Background/Context domain. However, the Blinding and Follow-up domains were not included because items related to randomisation, blinding or follow-up are not relevant to research into place of birth. Following this process of comparison, two items were added to the final version (items 1 and 9 in Fig 1).

**Table 4:** Comparison of ResQu Items with domains of study quality.

Deeks et al evaluation^1^			ResQu Index (final version)	
No.	Domain	Item	No.	Item
1	**Background / context**	Provision of background info		-
		Question clearly stated	1	Clear statement of research question
		Study originality		-
		Relevance to clinical practice		-
		Rationale/theoretical framework		-
2	**Sample definition and selection**	Retrospective/prospective	3	Type of study design
		Inclusion/exclusion criteria	19	Consistent inclusion criteria
		Sample size	712	Sample size calculationSample size power
		Selected to be representative	8	Reliable sampling, recruitment
		Baseline characteristics described	217	Defines each BSCharacteristics of cohorts
3	**Interventions**	Clear specification	2	Defines each BS
		Clear specification	11	Provider indicated, measured
		Concurrent/concomitant treatment		NRBS
		Feasibility of intervention		NRBS
		-	10	BS identified at appropriate time in pregnancy
4	**Outcomes**	Clear specification	4	Defines key terms, outcomes
		Objective and/or reliable		-
		Selected for relevance, importance, side-effects		-
5	**Creation of treatment groups**	Generation of random sequence		NRBS
		Concealment of allocation		NRBS
		How allocation occurred		NRBS
		Any attempt to balance groups by design	17	Cohorts with comparable characteristics
		Description of study design	3	Type of study design
		Suitability of design		-
		Contamination	6	Distinguishes between planned/unplanned HB
6	**Blinding**	Blind/double blind allocation		NRBS
		Blind outcome assessment		NRBS
		Maximum potential blinding used		NRBS
		Testing of blinding		NRBS
7	**Soundness of information**	Source of information about intervention	2	Defines and describes each birth setting clearly
		Source of information about outcome	9	Outcome data from reliable source
8	**Follow-up**	Equality of length of FU for two groups		NRBS
		Length of FU adequate?		NRBS
		Completeness of FU		NRBS
9	**Analysis: comparability**	Assessment of baseline comparability	19	Consistent inclusion criteria
		Assessment of baseline comparability	2017	Controls for confoundersCohorts with comparable characteristics
		Assessment of baseline comparability	16	Missing data reported and minimised
		Identification of prognostic factors		-
		Case mix adjustment		-
10	**Analysis: outcome**	Intention to treat analysis	18	Intention to treat analysis
		Appropriate methods of analysis	15	Reliable stats methods
		Pre-specified hypothesis		-
11	**Interpretation**	Appropriately based on results	22	Conclusions based on reported results
		Assessment of strength of evidence	23	Impact of cohort size
		Application/implications		-
		Clinical importance and statistical significance		-
		Interpretation in context	26–27	Regional variations in protocols and integration
12	**Presentation and reporting**	Completeness, clarity and structure		-
		Statistical presentation and reporting	21	Comparisons presented clearly and effectively
		Statistical presentation and reporting	23–25	Limitations
	-	-	13–14	Transfer between BS and timing indicated
	-	-	5	Ethics approval

^1^ See Deeks et al 2003 [39].

**Abbreviations:** BS = birth setting, HB = home birth, NRBS = not relevant to birth setting research.

Shaded items are specific to birth setting

Three ResQu items (items 10, 13 and 14 in Fig 1) are not compatible with any of the domains identified, as they constitute birth setting-specific items i.e. timing of decision about birth place and instances where women transfer from home or birth centre to give birth in hospital. Item 6 is also unique to birth place research, and was included in the Index because it identifies a potential source of bias.

We also compared the Index to the domains identified in the Cochrane ROBINS-I tool [38] devised to assess risk of bias (Table 5).

**Table 5:** Comparison of ResQu Items with domains in Cochrane risk of bias too (ROBINS-I).

Risk of bias domain (ROBINS-I)^1^	ResQu item/s	Comments
1. Bias due to confounding	20	It is unlikely that confounders will have no effect on outcomes. Potential impact addressed in Q20.
	10	Aims to reduce the impact of discontinuations and switches of BP for reasons that might be prognostic of outcome.
		Post intervention variables NA
2. Bias in selection of participants into the study	17–19	Q17 and Q19 aim to ensure comparable cohorts of participants. In BP studies selection into the study is not influenced by characteristics observed after start of intervention. Q18 ensures studies maintain participants in appropriate cohorts (intention to treat).
	4	Addresses whether risk levels are clearly defined
3. Bias in classification of interventions	2, 6, 11	Q2 ensures interventions (BP) are clearly defined; Q11 addresses clarity about provider type (part of intervention). Q6 ensures that HB cohorts are clearly and accurately defined as planned HB with skilled birth attendants i.e. more comparable with planned hospital and BC births.
4. Bias due to deviations from intended interventions	6, 10, 18	Adherence to an intention to treat analysis (Q18) ensures that outcomes are attributed to planned rather than actual BS. Q6 and Q10 further ensure that participants are linked appropriately to BP cohorts.
	13, 14	Take account of changes in intervention, i.e. transfer from HB or BC to hospital
5. Bias due to missing data	16, 24	Ensure that studies minimise incomplete data and address the impact of missing data on outcomes.
6. Bias in measurement of outcomes	9, 12–16, 21, 23	While it is impossible to blind participants and providers to intervention (BP), these questions ensure that outcomes are assessed carefully across cohorts. Use of reliable data sources (Q9) may prevent measurement errors. Q12 and Q23 address the implications of study design for rare outcomes (e.g. mortality). Q13 and Q14 ensure that outcomes for maternal transfer are reported accurately. Q15 addresses comparisons of outcomes between cohorts and Q21 how effectively these are reported.
7. Bias in selection of the reported results	21, 22	ResQu aims to ensure that results are reported accurately and clearly in relation to the stated research question (Q1). It does not address the selection of outcomes reported from the wider pool of study results generated, nor the selection of subgroups for which results are presented.

^1^ See Sterne et al 2015. [38]

**Abbreviations:** BC = birth centre, BP = birth place, HB = home birth, NA = not applicable, Q = question (item).

### Pilot testing

The pilot testing phase was conducted concurrently with a systematic review of literature on outcomes by place of birth, where the ResQu Index scores were used to assess the research quality of studies that met the review’s inclusion criteria. This process confirmed its utility, feasibility and applicability in a systematic review process. We were able to generate scores for each reviewed study, with congruence between reviewers, and excluded studies scoring less than 75% in the sensitivity analyses.

Team members also recorded how long it took them to read and assess a sample of 24 articles using the ResQu Index. This ranged from 15 to 45 minutes per article depending on article length, clarity and detail, with a mean of 31.25 minutes per article. The scoring process became quicker over time, as raters became more familiar with the items and scoring rubrics.

The pilot testing phase highlighted a few limitations in the Index and suggested potential new items or, more commonly, new levels or examples within the scoring rubric. We discussed discrepancies in scores until these could be resolved by team consensus; we also used this experience to refine the Index by clarifying the wording of items and scoring rubrics. The final Birth Place ResQu Index appears in Fig 1.

## Discussion

We undertook a formal, rigorous process to develop and validate a Birth Place Research Quality Index that provides a reliable means of assessing the quality of research on place of birth. The ResQu Index provides a consistent, transparent, and pragmatic solution to the dilemma presented by the ongoing scientific debate on the significance of results from studies on birth place [12]. The inclusion of 21 experts from various disciplines in the development and content validation process has added to the scope, acceptability, and utility of the Index [55]. Our collaborative approach involved ongoing correspondence and exchange with several experts, to ensure applicability for researchers and clinicians across the health professions. The S-CVI scores, close to or above .90, indicate excellent content validity as assessed by this large panel of international, multi-disciplinary experts.

The resulting ResQu Index is a more comprehensive, nuanced, and workable tool to assess the relative significance of studies than checklists which simply identify whether research adequately incorporates certain essential elements [40]. While the ResQu Index includes items fundamental to research integrity, such as clarity of objectives, appropriate sampling, treatment of confounders and use of statistical analysis [60], it also requires users to consider issues specific to childbirth and potentially attributable to place of birth. Moreover, the formal content validation process reflects expert consensus on wording and parameters of items to be included, as well as scoring and weighting of each aspect.

The compatibility of the ResQu items with most research quality domains [39] (Table 4) and with the ROBINS-I framework [38] (Table 5) indicates the comprehensiveness of the Index and its capacity to evaluate research rigour according to commonly-accepted standards. It excludes items not pertinent to birth place research, such as randomisation and blinding. Conversely, it does address critical items to assess quality of studies on birth place, such as maternal transfer, and ensuring that data on planned place of birth are not tainted by unintended home births or those without a qualified birth attendant [43–46].

The ResQu Index identifies birth place as the ‘intervention’; however, childbirth is a unique phenomenon within healthcare because birth place cannot be considered in terms of ‘dose’ or ‘exposure’. Issues of withdrawal or drop-out from the intervention have very distinct implications within a study on birth setting and must be treated differently from withdrawal in studies of other interventions. The process of developing the items required attention to the distinction between the quality of a study’s methods and the quality of reporting results [61]. This generated discussion within the project team during the development and pilot stages about the extent to which we were rating the research or the article.

The final stage of developing the ResQu Index was to use it in a systematic review to assess the quality of studies (published between 2000 and 2016) on the maternal and perinatal outcomes of different places of birth, for women with healthy, low-risk pregnancies in high-income countries. Two authors separately and jointly used the ResQu Index system to inform a rigorous process of inclusion and exclusion of identified studies. They also developed a user dictionary (available on request) to assist with using the Index to assess studies in practice. While several low-scoring studies were excluded by the parameters of our systematic review (e.g. because they did not follow an intention to treat approach, or they included births to women at different levels of risk), we were able to utilise the Index to examine the quality of studies to inform inclusion in our meta-analysis of data.

Place of birth research has been plagued with controversy over study design, variables to be measured, cohort definition and other factors specific to this context. A growing volume of academic literature and media attention has focused on research about birth place. The availability of a reliable method to assess quality of the evidence has the potential to create consensus about the quality of studies among clinicians and researchers who have been (often fiercely) divided on the issue [12, 62, 63]. Women and families are ill-served by inter-professional conflict, given that their choices are influenced by provider attitudes [11, 32, 64, 65]. In the age of increased consumer access to scientific findings, it is imperative that public health information and recommendations are based on the best available data. Studies that score higher on the Index thus have greater potential to reliably inform the evidence base for decisions about birth place by women and health professionals.

The ResQu Index could facilitate the development of clinical guidelines on provision of maternity services across places of birth. For example, following a review of the evidence, some professional clinical bulletins [28] have relied on conclusions from publications that are widely cited [22] but have been subject to critical reviews questioning their quality [44, 46]. Other investigators have published ethical practice recommendations based on findings from research that would not meet the ResQu Index scoring threshold to support a recommendation [62]. Consumers and clinicians will benefit from having a consistent, transparent and reliable method to distinguish and rate the relative importance of research conclusions and recommendations related to place of birth. In addition to guiding the design of studies, the ResQu Index could provide a framework for peer review for publication or serve as a quality assurance guide for research grant funding agencies. The Index could also be used to appraise research on birth place across population cohorts, taking adequate account of the characteristics of women in different settings, and applying the same principles of comparability between cohorts and transparency of research methods. Finally, the Index may help to contextualise results of research in jurisdictions where care is not integrated across settings.

### Strengths and limitations

The development of a quality assessment instrument is a complex process, benefiting from the contributions of a multidisciplinary team. Despite careful measures to maximise content validity and consistency, it inevitably involves subjective judgements, both in the selection and weighting of items, and their application to appraise published studies of place of birth. Other researchers may debate its relative emphasis on some aspects of this literature at the expense of others.

The results of pilot-testing confirm that the Index is, like other such scales, subject to some personal interpretation. However, comparison of the inter-rater consistency and consensus of scores from two raters, including one non-clinician researcher, with a large selection of studies showed considerable similarity. One of the strengths of the Index is its capacity to facilitate multi-disciplinary, collaborative evaluation of research outputs, by using commonly-defined terms.

The ResQu Index was not designed to assess qualitative studies and would not, by virtue of its focus on specific aspects of methodology, rate them highly. We acknowledge that some important questions about quality and safety of birth place, or person-centred care, cannot be answered by quantitative methods alone.

## Conclusions

The Birth Place Research Quality Index is a reliable, pragmatic tool to systematically appraise the quality of research on place of birth. The composite scoring system highlights the unique nature of childbirth and the specific characteristics of study design and analysis that must be considered when evaluating reported findings. We used a detailed, formal process to design and content validate the Index in consultation with an international panel of multi-disciplinary experts in this field. Pilot testing demonstrated usability, feasibility, and inter-rater consistency. The Index was well suited to systematic review of research on birth place. It could also be used to inform clinical guidelines on maternity service provision across birth settings, to inform the design of studies, or to provide a framework for peer review for publication.

## Supporting information

S1 Table Examples of how item wording changed following expert review. (DOCX)

S2 Table Examples of how rating of items changed following expert review. (DOCX)
