# Supplementary material for: The ResQu Index: A new instrument to appraise the quality of research on birth place
Source: PLoS One. 2017 Aug 10;12(8):e0182991. doi: 10.1371/journal.pone.0182991 (PMC5552354; doi:10.1371/journal.pone.0182991)
Supplement: S1 Table — (DOCX) [file pone.0182991.s001.docx]

**Table S1: Examples of how item wording changed following expert review**

| **Item on characteristics of groups compared** | |
| --- | --- |
| Original wording | *3. Uses reliable and logical comparison groups* |
| CVI scores | Clarity = .76  Relevance = .95  Importance = 1.00 |
| Expert reviewer comments | ‘Item 3 might benefit from some detail on what constitutes a reliable and logical comparison group.’  ‘Item is clear, but would require explanatory notes to accompany them to make sure scores are allocated on the same basis by all users.’  ‘Aspects of a reliable and logical comparison group should be listed.’ |
| Research team response | Experts clearly felt this item was vital to the Index but considered that the wording was unclear or potentially subjective (what is ‘logical’?), and did not express the importance of identifying an appropriate comparison group. The revised rating scale will also enhance clarity. |
| Revised wording and rating scale | *16. Uses cohorts with comparable obstetric and socio-demographic characteristics*  *0=no comparison group*  *1=cohorts differ in terms of risk*  *3=cohorts comparable by risk, but vary by socio-demographic characteristics*  *4=comparable socio-demographic and risk profile*  *6=cohort characteristics are matched* |
| **Items about transfer to hospital from other intended birth setting** | |
| Original wording | *12. Uses reliable method to track women when birth setting changes* |
| CVI scores | CVI scores: Clarity = .90, Relevance = 1.00, Importance = 1.00 |
| Original wording | *15. Reports criteria for transfer (change of birth place)* |
| CVI scores | CVI scores: Clarity = 1.00, Relevance = .90, Importance = .62 |
| Original wording | *16. Considers potential effects related to timing of transfer and delays to treatment* |
| CVI scores | CVI scores: Clarity = .81, Relevance = .95, Importance = .86 |
| Original wording | *17. Accounts for effect of mode of transfer (ambulance, private car, neonatal transport team)* |
| CVI scores | CVI scores: Clarity = .71, Relevance = .62, Importance = .48 |
| Expert reviewer comments | ‘#17 - not sure it is necessary to control for effects of these transport methods, but they should be reported and discussed in the context of service integration.’  ‘If the study includes home births, an attempt to understand and/or control for the travel time between the woman's home and the hospital.’  ‘I am really not sure about controlling for transfer times as this is part of the package when an out of hospital (and off site) setting is chosen so we need to know the outcomes that result with whatever transfer time occurs.’ |
| Research team response | Experts considered that some of these questions on transfer were not relevant to the Index, nor as important as other items. The treatment of transfers is complex, especially as it may occur at different stages before, during or after labour. Few studies indicate transfers for women intending hospital births. Item 12 was slightly reworded to ‘uses reliable method to indicate changes in birth setting’. Original items 15 and 17 were omitted, and item 16 reworded with simplified rating categories. |
| Revised wording | *12. Uses reliable method to indicate changes in birth setting*  *13. Indicates timing of transfer between birth settings within labour, birth or immediate postpartum period (Originally item 16).* |
