# Supplementary material for: The ResQu Index: A new instrument to appraise the quality of research on birth place
Source: PLoS One. 2017 Aug 10;12(8):e0182991. doi: 10.1371/journal.pone.0182991 (PMC5552354; doi:10.1371/journal.pone.0182991)
Supplement: S2 Table — (DOCX) [file pone.0182991.s002.docx]

**Table S2: Examples of how rating of items changed following expert review**

| **Item on impact of provider type (midwife, obstetrician) on outcomes** | |
| --- | --- |
| Original wording and rating scale | *7. Accounts for effect of provider type*  *0=no recognition of effect 2=acknowledged but not accounted for 4=accounts fully for effect of provider 6=compares same providers across settings* |
| CVI scores | Clarity = .67  Relevance = .90  Importance = .81 Ratings score = .60 |
| Expert reviewer comments | - |
| Research team response | Experts considered that this item was relevant and important to the Index but unclear especially in terms of the proposed rating scale. |
| Revised wording and rating scale | *10. Provider type (for birth) is indicated, measured and adjusted for in analysis*  *0=provider type not indicated or measured 1=provider type reported 2=provider type indicated but no stratification or adjustment for provider type 3=analysis stratified by provider type 4=provider type adjusted for in analysis OR  4=NA i.e. single provider type across study.* |
| **Item about level of integration between different birth settings** | |
| Original wording and rating scale | *13. Addresses effect of level of service integration between home, birth centre and hospital*  *0=no 2=acknowledges effect 4=adequately accounts for effect of integration 6=fully integrated system* |
| CVI scores | Clarity = .86  Relevance = .86  Importance = .90 Ratings score=.70 |
| Expert reviewer comments | ‘Item 13 seems problematic. Rather than penalize a study's quality based upon integration characteristics of the health-care system in which the study is done, this information needs to be acknowledged and tracked.’  ‘A concern I have about this scale is that it may penalize research from regions where home birth and out-of-hospital birth is less formally recognized, less integrated into healthcare systems, and less socially/culturally normalized’ |
| Research team response | We recognise that the Index cannot effectively address the broader issues about the difficulties of conducting such research in regions where home and birth centre births are more widely accepted and therefore better documented, thereby facilitating research into birth setting. |
| Revised wording and rating scale | *25. Addresses effect of level of service integration between home, birth centre and hospital*  *0=no 2=acknowledges effect of limited integration 4=accounts for effects of limited integration OR 4=well-integrated system* |
